# Fluorescent Silver Nanoclusters Associated with Double-Stranded Poly(dGdC) DNA

**DOI:** 10.3390/nano15050397

**Published:** 2025-03-05

**Authors:** Zakhar Reveguk, Roberto Improta, Lara Martínez-Fernández, Ruslan Ramazanov, Shachar Richter, Alexander Kotlyar

**Affiliations:** 1 Department of Materials Science and Engineering, The Iby and Aladar Fleischman Faculty of Engineering, University Center for Nanoscience and Nanotechnology, Tel Aviv University, Tel Aviv 6997801, Israel; zakhar@mail.tau.ac.il; 2 The George S. Wise Faculty of Life Sciences, Tel Aviv University, Tel Aviv 6997801, Israel; 3 Istituto di Biostrutture e Bioimmagini-CNR (IBB-CNR), Via De Amicis 95, I-80145 Napoli, Italy; robimp@unina.it; 4 Departamento de Química, Facultad de Ciencias and Institute for Advanced Research in Chemical Science (IADCHEM), Universidad Autónoma de Madrid, Campus de Excelencia UAM-CSIC, Cantoblanco, 28049 Madrid, Spain; lmartinez@iqf.csic.es; 5 Department of Chemistry, University of Helsinki, FI-00014 Helsinki, Finland; ruslan.ramazanov@helsinki.fi

**Keywords:** DNA, silver nanoclusters, fluorescence, AFM, HRTEM, quantum mechanical calculations

## Abstract

Here, we demonstrate through AFM imaging and CD spectroscopy that the binding of silver ions (Ag^+^) to poly(dGdC), a double-stranded (ds) DNA composed of two identical repeating strands, at a stoichiometry of one Ag^+^ per GC base pair induces a one-base shift of one strand relative to the other. This results in a ds nucleic acid-Ag^+^ conjugate consisting of alternating CC and GG base pairs coordinated by silver ions. The proposed organization of the conjugate is supported by the results of our Quantum Mechanical (QM) and Molecular Mechanics (MMs) calculations. The reduction of Ag^+^ ions followed by the partial oxidation of silver atoms yields a highly fluorescent conjugate emitting at 720 nm. This fluorescent behavior in conjugates of long, repetitive ds DNA (thousands of base pairs) with silver has never been demonstrated before. We propose that the poly(dGdC)–Ag conjugate functions as a dynamic system, comprising various small clusters embedded within the DNA and interacting with one another through energy transfer. This hypothesis is supported by the results of our QM and MMs calculations. Additionally, these DNA–silver conjugates, comprising silver nanoclusters, may possess conductive properties, making them potential candidates for use as nanowires in nanodevices and nanosensors.

## 1. Introduction

Silver nanoclusters (AgNCs) are small nanostructures typically composed of several metal atoms and ions [1]. The photophysical properties of the clusters depend on their size [2], charge, and geometry [3], as well as on the properties of the surrounding environment [4,5]. The metal core of hydrophilic Ag-NCs is coated with water-soluble ligands [6,7,8], including DNA [9]. Some DNA-templated silver nanoclusters (DNA-AgNCs) exhibit fluorescence with high quantum yields (QYs) due to the specific interactions between silver ions and the DNA scaffold [10,11]. The clusters are commonly synthesized through a two-step process: conjugating relatively short (10–40 bases) DNA oligonucleotides with silver ions (Ag^+^) and reducing the ions with a strong reductant [11]. Due to their high affinity, silver ions bind to the nucleic acid bases rather than to the backbone [12,13,14]. The affinity of the ions to nucleic bases is relatively high and decreases in the following order: C, G, A, and T [15]. In DNA, the ion primarily binds through the N7 position of guanine [16] and the N3 position [17] of cytosine. Incubation of DNA-bound silver ions with a strong reductant leads to the complete and rapid reduction of the ions to the metal atoms (Ag^0^) [18]. Conjugates of reduced silver with DNA do not exhibit fluorescence, regardless of the DNA sequence. However, subsequent oxidation of some DNA-associated silver atoms by molecular oxygen induces fluorescence in the conjugates [19]. High-resolution mass spectrometry (HRMS) analysis demonstrated that in HPLC-purified DNA-AgNCs approximately half of the silver atoms maintain their cationic state [2,20]. The interaction of silver ions with nucleic bases plays a crucial role in determining the composition and properties of oligonucleotide-associated clusters [20,21,22]. A wide range of DNA-AgNCs, composed of various oligonucleotide sequences and comprising varying numbers of silver atoms and ions, have been described [23,24,25]. These clusters exhibit different fluorescence parameters. Their emission spectra span the entire visible (VIS) and near-infrared (NIR) regions [10,21,26]. Additionally, interactions between clusters through energy transfer have been reported [4,27]. DNA-AgNCs are chiral and display robust circular dichroism (CD) signals [28,29], enabling the extraction of information about their structure through CD spectroscopic analysis. Over the past decade, significant progress has been achieved in understanding the correlation between fluorescent properties of DNA-AgNCs and their composition (number of silver atoms, DNA length, and sequence) and 3D structure. Recently, crystal structures of several highly fluorescent DNA-AgNCs have been reported, providing insights into the structural basis of the cluster’s fluorescence [10,30]. These data, combined with theoretical knowledge, should pave the way for designing novel DNA-associated Ag-NCs with tailored photophysical properties. Notably, most studies on DNA-AgNCs have focused on single-stranded (ss) DNA, typically composed of 5–40 nucleotides. Only a few studies on AgNCs associated with double-stranded (ds) DNA containing one or a small number of mismatches have been published [31,32]. To our knowledge, no studies have been published so far on fluorescent conjugates of long (up to thousands of base pairs) double-stranded (ds) repeating DNA.

Here, we report the formation of a stoichiometric complex between silver ions and long (ranging from hundreds to thousands of bases) (ds)DNA composed of two strands of the same alternating GC sequence, poly(dGdC). Based on AFM imaging analysis, CD spectroscopy, and theoretical calculations, we conclude that the binding of silver ions causes a one-base shift of a DNA strand relative to the other, yielding the ds polymer composed of alternating C-Ag^+^-C and G-Ag^+^-G metal-stabilized base pairs. The reduction of the bound silver ions, followed by slow (hours) partial oxidation of the atoms, induces fluorescence with maximum excitation at 280/620 nm and maximum emission at 720 nm. Based on experimental data and theoretical calculations, we propose that the observed fluorescence of the poly(dGdC)–Ag conjugate is due to clusters composed of a few silver atoms and ions.

## 2. Materials and Methods

### 2.1. Materials

Unless otherwise stated, the reagents were obtained from Sigma-Aldrich (St. Louis, MO, USA) and were used without further purification. Polymerase I (PolyI) and Klenow fragment exonuclease of the polymerase lacking the 3′→5′ exonuclease activity (Klenow exo−) were purchased from Lucigen Corporation (Middleton, WI, USA). Oligonucleotides were purchased from Integrated DNA Technologies, Inc. (Integrated DNA Technologies, B.V., Leuven, Belgium) unless stated otherwise.

### 2.2. DNA Synthesis

The DNA synthesis was conducted essentially as described elsewhere [33]. The synthesis of 150 b.p. poly(dGdC) was conducted in 60 mM Tris-HCl (pH 7.5), 3.2 mM MgCl_2_, 5 mM dithiothreitol 1 mM dGTP, 1 mM dCTP, 4.5 µM 40 base pair (bp) poly(dGdC), and 0.16 U/µL PolyI for about 16 h at 37 °C. To remove the enzyme and other components of the assay, the synthesized molecules were chromatographed on a 10 mL Sepharose CL-2B column (1 × 5 cm) equilibrated with 10 mM HEPES-K (pH 7.5). The void volume fraction was collected and stored at 4 °C until used. The synthesis of 1500 bp poly(dGdC) was achieved by extending 0.2 µM 150 bp poly(dGdC) molecules by 0.16 U/µL Klenow exo− in the same assay mixture for approximately 16 h at 37 °C. The resulting product was chromatographed as above.

### 2.3. Purification of Poly(dGdC) Molecules

To obtain highly uniform molecules, the synthesized poly(dGdC) was purified from the nucleic acid molecules of various lengths using a 1.5% agarose gel. The DNA was electrophoresed for about an hour at 110 V. Subsequently, the gel piece containing the DNA was cut out from the gel with a razor blade, placed into a dialysis bag (10k MWCO) and the DNA was electroeluted. The electroeluted DNA was incubated with Q Sepharose High Performance beads equilibrated with 10 mM HEPES-K (pH 7.5) and 100 mM KNO_3_ for 15 min. The beads with bound DNA were then centrifuged at 2000 rpm for 5 min on a benchtop centrifuge. To detach the DNA, the beads were suspended in 10 mM HEPES-K (pH 7.5) and 1.5 M KNO_3_ and incubated for 15 min under ambient conditions. The beads were centrifuged at 2000 rpm for 5 min on a benchtop centrifuge, and the supernatant was chromatographed on a Sepharose CL-2B column (1 × 5 cm) equilibrated with 10 mM HEPES-K (pH 7.5). The void volume fraction was collected and used.

### 2.4. Preparation of DNA-Ag^+^ and DNA-AgNC Conjugates

To a 30–100 µM solution (in base pairs; OD at 260 nm is 0.5–1.5) of poly(dGdC), a small volume (several microliters) of 10 mM AgNO_3_ solution was added to achieve an Ag^+^ concentration equal to that of the GC pairs. The mixture was then vigorously stirred and incubated for 5 min under ambient conditions. Subsequently, several microliters of freshly prepared 10 mM NaBH_4_ solution were added under vigorous stirring to achieve a final concentration of 100–200 µM. The reduced sample was kept at room temperature in the dark for 24 h and then stored at 4 °C.

### 2.5. Atomic Force Microscopy (AFM)

A 100 µL drop of a DNA solution in 10 mM MgAc was applied onto freshly cleaved mica and allowed to sit on the surface for 5 min. The surface was subsequently rinsed with cold double-deionized MQ water (DDW) and dried with an N_2_ flow. AFM imaging was conducted using a Solver PRO AFM system (NT-MDT, Zelenograd, Russia) in semi-contact mode, using 130 µm-long Si-gold-coated cantilevers (ScanSens, Munich, Germany) with a resonance frequency of 70–180 kHz. The images were “flattened” (each line of the image was fitted to a second-order polynomial, and the polynomial was then subtracted from the image line) with Nova image processing software (Version 3.5, NT-MDT, Moscow, Russia) and analyzed using Gwyddion software (http://gwyddion.net/, accessed on 22 February 2025).

### 2.6. Extending AgNCs in a Poly(dGdC)–AgNC Conjugate

Poly(dGdC)–AgNC conjugates were deposited on mica following the procedure described in Section 2.5. The surface was then rinsed with 20 mM magnesium acetate (Mg-Ac) and kept moist, leaving a small volume (10–30 µL) of Mg-Ac solution. Next, 100 µL of the extending solution, containing gold salt and ascorbic acid, was applied. Gold ion reduction by ascorbic acid preferentially occurred on AgNC seeds, promoting their growth. The extending solution was prepared by mixing 74 µL of DDW with 25 µL of 10 µM HAuCl_4_ and 1 mM KBr, followed by incubation at ambient temperature for at least 30 min. Just before deposition on mica (within 1–2 s), 1 µL of freshly prepared 100 mM potassium ascorbate, obtained by neutralizing ascorbic acid with an equimolar concentration of KOH, was added.

### 2.7. Absorption, CD, and Fluorescent Spectroscopy

The UV-Vis absorption spectra measurements were carried out under ambient conditions with a Scinco S-3100 spectrophotometer (Seoul, Republic of Korea) The CD spectra were recorded with a Chirascan V100 CD spectrometer (Applied Photophysics, Leatherhead, UK). The fluorescence spectra were recorded using RF-6000 (Shimadzu, Kyoto, Japan) Slits 5/5 nm for excitation/emission. Time-resolved kinetic fluorescence experiments were conducted using Fluorolog-3 (HORIBA, Kyoto, Japan) equipped with the NanoLED 650-L (HORIBA). Decay curves were fitted using the DecayFit tool (FluorTools, Seoul, Republic of Korea).

### 2.8. High-Resolution Transmission Electron Microscopy (HRTEM) Samples Preparation and Measurements

The DNA–silver conjugates (1.5 µL) were mixed with 10 µL of 20 mM Mg(NO_3_)_2_ and deposited on a carbon grid (LC200-CU-CC from Electron Microscopy Sciences, Hatfield, Montgomery County, PA, USA) treated with 25% O_2_ and 75% Ar plasma before deposition. The carbon surface was rinsed with DDW 3–5 min after the deposition, dried under low vacuum for 15 min, and treated with 25% O_2_ and 75% Ar plasma. Measurements were conducted on a TALOS (ThermoFisher Scientific, Waltham, MA, USA) in 200 kV bright field mode.

### 2.9. Quantum Mechanical Calculations of CD Spectra

Various structural models were optimized starting from two available crystallographic structures of Ag^+^ containing oligonucleotides [34,35]. We employed Quantum Mechanical (QM)/Molecular Mechanics (MMs) calculations where the nucleobases and the Ag^+^ ions were treated at the QM level, and the backbone and external counterions were considered at the MMs level. Density Functional Theory (DFT) was used as the QM method with the M052X [36,37] functional, employing LANL2DZ pseudopotential for Ag^+^, and the def2-svp basis set for the other atoms [38]. The latest implementation of the AMBER force field was used for the MMs part. As detailed in Supplementary Materials, full QM test calculations were also performed, yielding results similar to those of QM/MMs calculations. Once the ground state geometry was optimized, we computed the absorption and CD spectra using the time-dependent version of DFT. The solvent effect has been included using the Polarizable Continuum Model [39] (PCM). This approach has been successfully employed in studying the spectral properties of oligonucleotides [40,41], including those coordinating Ag^+^ ions [42,43]. All these calculations have been performed by using the Gaussian 16 program [44]. Additional calculation details are reported in the Supplementary Materials (Figures S1–S6).

### 2.10. Quantum Mechanical Calculations of the DNA Luminescence

To analyze the luminescent characteristics of silver atoms embedded in the poly(dGdC) double helix, we employed a two-level subtractive combined model QM1/QM2. This model describes the optical properties of partially oxidized silver clusters, assuming that 5s^1^ electrons contribute to the optically active S_1_ state, while 4d electrons establish bonds with stabilizing ligands [45]. In our calculations, we have been focused on the excited S_1_ state, primarily associated with metal atoms. Thus, the entire systems in our approach were divided into two parts, with distinct levels of description. A similar combined QM and MMs approach was recently evaluated for modeling the optical properties of a DNA-templated silver cluster [46]. At the high QM1 level, silver atoms were described using the DFT PBE0 functional [47] and the def2-tzvp basis sets [48], while at the low QM2 level, the nitrogenous bases and the sugar-phosphate backbones were described using the semi-empirical tight-binding model GFN2-xTB (XTB2) [49], which has proven to be efficient for calculating non-covalent interactions in large molecular systems. Under these conditions, the geometry optimizations of the ground and lowest singlet excited states were carried out. All calculations were conducted using the ALPB continuum water model [50] in the ORCA 5.0.4 program [51]. To analyze the redox potential, we calculated the change in the free energy of ionization for silver atoms surrounded by a pair of cytosines and guanines using the Born-Haber cycle [52]. These redox calculations were performed using density functional theory at the PBE0/def2-tzvp level and the polarizable continuum model (PCM) [39] within the Gaussian16 program [44].

## 3. Results and Discussion

**Figure 1:**
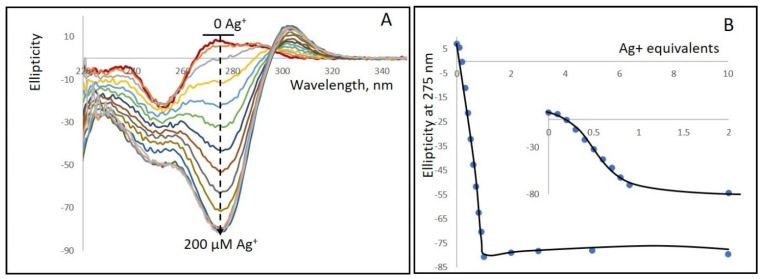
(**A**) CD spectra of a 1kbp poly(dGdC) conjugated with Ag^+^ at various DNA (bp) to Ag^+^ ratios. A 20 µM (in bp) DNA solution (red curve at the top) was incubated for 10 min at 25 °C with: 2, 4, 6, 8, 10, 10, 12, 14, 16, 18, 20, 40, 60, 100, and 200 µM AgNO_3_. The addition of silver ions results in the appearance of a strong negative band centered around 275 nm (black dashed curve) and a weaker positive band with a maximum at 303 nm. (**B**) Dependence of the CD signal amplitude at 275 nm on Ag^+^ concentration. The spectra were measured as described in Section 2. The inset shows an enlarged view of the 0 to 40 µM Ag^+^ range.

Figure 1 and Figure S7 demonstrate that poly(dGdC) binds silver ions (Ag^+^) tightly with a 1:1 stoichiometry of Ag^+^ to base pairs, resulting in significant changes in both the DNA’s CD spectra and absorption spectra, respectively. Upon silver ion binding, the absorption increases in the 270–310 nm range while decreasing in the 240–260 nm range (Figure S7A,B). Figure S7C shows that the absorption at 287 nm increases linearly with Ag^+^ concentration in the 0–60 µM range (DNA concentration expressed in bp and did not change with further increases in Ag^+^ concentration. These results indicate that the binding of one Ag^+^ per GC pair in poly(dGdC) leads to the observed changes in the spectrum.

The binding of silver ions to the DNA significantly alters the CD spectrum (Figure 1A,B). Upon addition of silver ions, a negative band centered around 275 nm appears, along with a positive band with a maximum at about 300 nm. The most pronounced spectral changes occur when the ion concentration is equivalent to the DNA concentration (expressed in base pairs). Increasing the ion-to-bp concentration beyond one does not lead to any noticeable changes in either the spectrum’s shape or the intensity of the CD signal at 275 nm. These observations indicate that, in the conjugate, one Ag^+^ is bound to each GC pair in the DNA.

**Figure 2:**
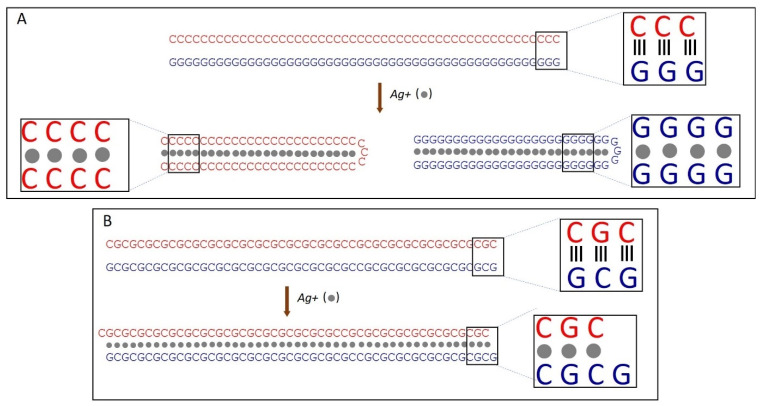
Scheme of Ag^+^-induced changes in poly(dG)-poly(dC) (**A**) and poly(dGdC) (**B**) molecules. (**A**) Binding of silver ions (grey spheres) to poly(dG)-poly(dC) leads to strand separation and subsequent folding of each strand into a hairpin structure. In these folded structures, the strand halves run antiparallel and hybridize with each other, forming either GG or CC base pairs stabilized by silver ions located in the core (between the bases) of the DNA. (**B**) Binding of silver ions (grey spheres) to poly(dGdC) causes one strand to shift relative to the other by one nucleotide. The structure is stabilized by the interaction of GG or CC base pairs with Ag-ions [53].

**Figure 3:**
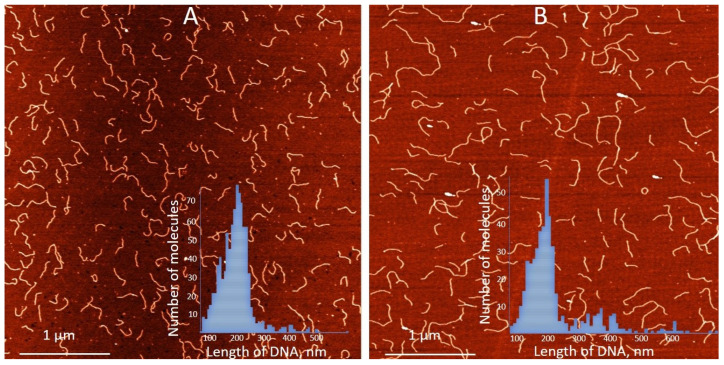
AFM images and contour length statistics of 700 bp poly(dGdC) molecules (**A**) conjugated with silver ions (**B**). The DNA and the conjugate were prepared as described in Section 2. Both types of molecules were deposited on mica under the same conditions. Statistical contour length analysis was performed on more than 200 single, well-separated molecules of each type. Insets in (**A**,**B**) show the statistical analysis of poly(dGdC) and the conjugate, respectively. The average length of poly(dGdC) and the conjugate are correspondingly equal to 214 ± 30 nm and 226 ± 35 nm.

We have previously demonstrated that the binding of silver ions to poly(dG)-poly(dC), composed of G- and C-homopolymer strands, causes the strands to separate [53]. Each strand then folds independently into structures composed of G-Ag^+^-G and C-Ag^+^-C base pairs, respectively [53] (see scheme in Figure 2A). This process involves each strand folding back on itself in a hairpin fashion and hybridizing with its own halve (Figure 2A). Consequently, the length of the resulting folded structures is approximately half that of the original poly(dG)-poly(dC) molecule [53]. AFM imaging reveals that Ag^+^ binding to poly(dGdC) does not affect the DNA’s contour length. As shown in Figure 3A,B, poly(dGdC)–Ag^+^ maintains the same length as unmodified poly(dGdC), within experimental error.

The height of poly(dGdC) also remains unchanged upon conjugation with silver ions (Figure S8). The lack of noticeable changes in the DNA structure, along with the thermodynamic preference for G-Ag^+^-G and C-Ag^+^-C pairs over a GC one [53] led us to propose that the binding of Ag^+^ ions causes one strand to slide relative to the other by one nucleotide (Figure 2B). This shift aligns each C base directly opposite another C base and each G base directly opposite another G base on the other strand (see Figure 2B). In the conjugate, each base pair (CC or GG) is stabilized by the interaction of two C or two G bases with a silver ion.

**Figure 4:**
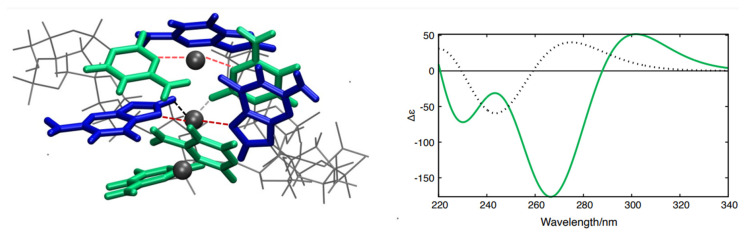
Structural model depicting GC pairs coordinating Ag^+^ ions (**A**) alongside the calculated ECD spectra (**B**) of a GC–Ag^+^ conjugate (green curve) and a bare GC pair in the B-DNA configuration computed using PCM/TD-M052X/AMBER QM/MMs simulations. The part treated at the QM level is depicted as the colored tube, and the MMs part as gray lines.

QM and QM/MMs calculations support this hypothesis. Three distinct structural models (Figure S3, see Supplementary Materials for details) based on X-ray crystal structures of GC oligonucleotides coordinating Ag^+^ ions [34,35] were constructed and optimized (Figure S5, see Supplementary Materials for details). We then computed the Electronic Circular Dichroism (ECD) spectra for each of the above models using an approach that has previously been successfully applied to simulate the spectral properties of DNA fragments (see computational details and Supplementary Materials). The spectral shape associated with one of the models (Figure 4, green curve in the inset) closely resembles the CD spectrum of the poly(dGdC)–Ag^+^ conjugate (Figure 1A) and notably diverges from the CD spectrum of a ‘standard’ GC fragment in B-DNA conformation (Figure 4, black dotted curve in the inset). In contrast, the ECD spectra corresponding to the other two models (see Supplementary Materials) significantly diverge from the experimental spectra, allowing us to focus on the former model. Similar to the spectrum of poly(dGdC)–Ag^+^ (Figure 1A), the computed ECD spectrum (Figure 4, green curve in the inset) also exhibits two distinct minima and a smaller positive maximum on the red side (at 300 nm). Based on the similarity between the computed and the experimental CD spectra and the X-ray structures [34,35], we hypothesize that Ag^+^ ions are coordinated by either CC or GG base pairs, adopting a distinct coordination geometry depicted in Figure 4. Notably, the N3 atoms of CC and N7 atoms of GG pairs serve as primary ligands for the Ag^+^, with the three atoms nearly aligned (interactions between the ions and the bases are shown by red dashed lines in Figure 4A). The CC or GG couples are significantly twisted around the N3-Ag-N3 axis, maximizing the interaction between carbonyl lone pairs and Ag^+^ ions (Figure 4A, black dashed lines). This arrangement results in two strands whose axes are tilted relative to each other, exhibiting a substantial propeller twist value (−50°~−70°) [43].

Based on the experimental and theoretical data, we conclude that the conjugate can be viewed as a chain of silver ions embedded within the DNA core, as depicted in Figure 4. Given that silver ions can be readily reduced and oxidized at relatively mild redox potentials, and the distance between neighboring ions is less than 1 nm, one can speculate that the conjugate might be capable of conducting electrical current along the nucleic acid polymer.

**Figure 5:**
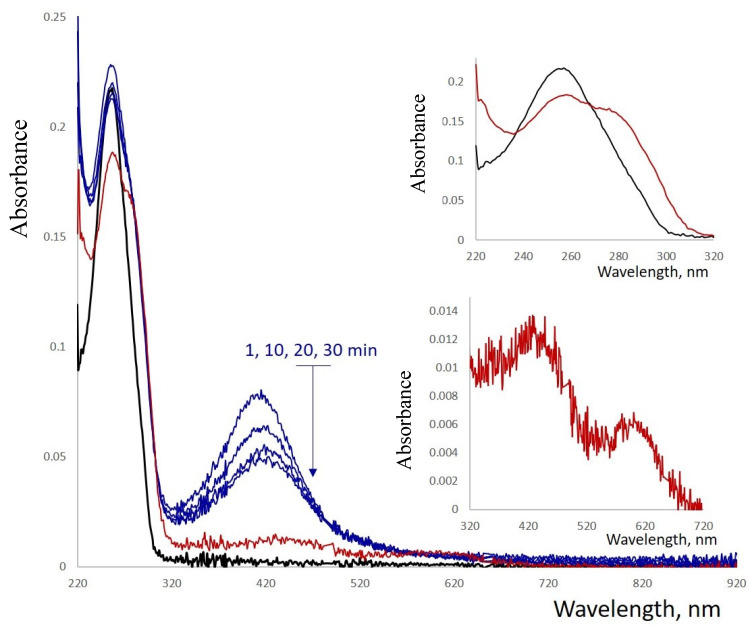
Absorption spectra of a reduced 1 kb poly(dGdC)–Ag conjugate. The DNA (black curve) was conjugated with silver ions at a stoichiometry of one silver ion per base pair and subsequently reduced with NaBH_4_ as described in Section 2. The spectra were recorded at 1, 10, 20, and 30 min (blue curves) and at 16 h (red curve) after the addition of the reductant. Insets show the magnified regions of the corresponding spectra in the 220–260 nm and 320–720 nm ranges.

Reduction of Ag^+^ by BH_4_^−^ resulted in changes in both the absorption and CD spectra of the conjugate (Figure S9). The shape of the spectra in the 230–300 nm region resembled that of poly(dGdC) not conjugated with silver. Additionally, an intensive band centered around 400 nm appears in the spectrum 5–10 min after the addition of the reductant (Figure 5). The intensity of this band decreases over time, accompanied by the appearance of a less intense absorption band with a maximum centered around 580 nm (Figure 5, see the inset at the bottom). The 425 nm band diminished within an hour, while the 580 nm band intensified, reaching maximum intensity after approximately 10 h.

**Figure 6:**
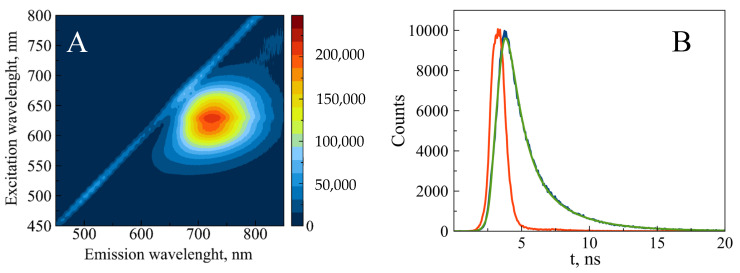
Steady-state (**A**) and time-resolved (**B**) fluorescence of partially reduced 700 bp poly(dGdC)–silver conjugate. The conjugate was prepared as described in Section 2. (**A**) The 2D fluorescence contour plot was recorded 24 h after reducing the DNA-Ag^+^ conjugate with BH_4_^−^. (**B**) The time-resolved kinetics were measured 24 h after reducing the DNA-Ag^+^ conjugate with BH_4_^−^ as described in Section 2. The instrument response function (IRF), measured signal, and bi-exponential fit are depicted as red, blue, and green curves, respectively.

The fully reduced conjugate is characterized by the 425 nm absorption band (Figure 5). The reduced form is non-fluorescent; however, partial oxidation induces fluorescence of the conjugate. The maximal intensity was observed at 620 nm excitation and 720 nm emission (Figure 6A). The fluorescence decay follows bi-exponential kinetics with lifetimes of 1.16 ns (80%) and 3.37 ns (20%) (Figure 6B). These values fall within the range observed for AgNCs associated with short ssDNA (1–10 ns) [24]. The fluorescence intensity remains unchanged for 3 months at 4 °C (Figure S10). The fluorescence and the absorption of the conjugate at 600 nm (see Figure 5) over several weeks.

**Figure 7:**
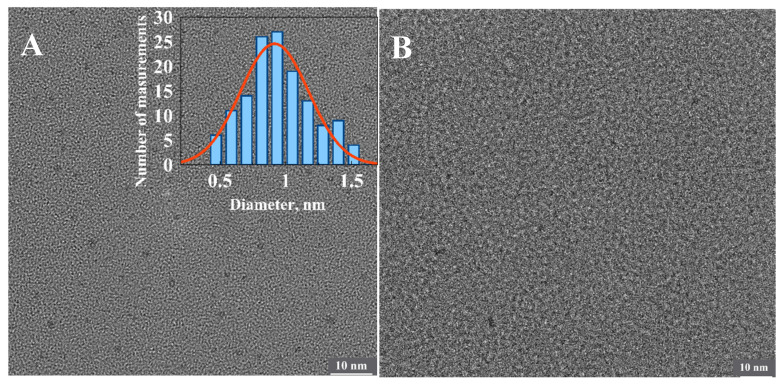
HRTEM images of the freshly reduced (**A**) and partly oxidized (**B**) conjugates of 1500 bp poly(dGdC) with silver. The inset shows a statistical analysis of the average diameter of more than 100 particles seen as dark dots in the image. The estimated diameter of the particles is equal 0.9 ± 0.3 nm.

We have made multiple attempts to identify the origin of the fluorescent clusters and the number of silver atoms comprising them using state-of-the-art HRTEM techniques. However, no detectable clusters associated with the partly oxidized 1500 bp-long fluorescent poly(dGdC)–Ag conjugate were observed on the surface (Figure 7B). However, the surface analysis revealed clusters approximately 0.9 nm in diameter associated with the completely reduced conjugate (Figure 7A). This indicates that the size of the initially formed DNA-templated reduced particles decreases upon oxidation. These tiny clusters also cannot be detected by AFM. AFM analysis revealed neither changes in DNA morphology nor the presence of embedded small particles (Figure S11). The contour length of the DNA was only slightly (by ~20%) reduced upon the reduction of silver ions in the conjugate (Figure S11). This reduction is likely due to the disruption of the regular ds structure of the conjugate caused by the cluster formation.

**Figure 8:**
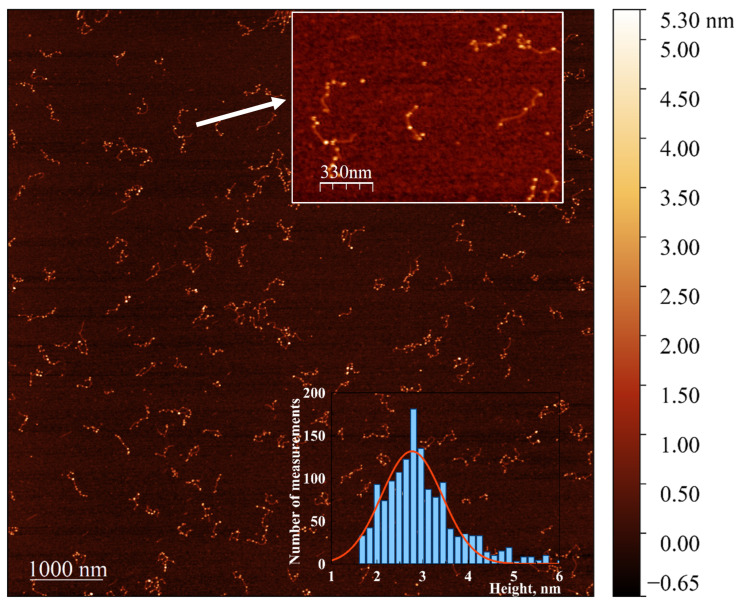
AFM image showing the extension of NCs in the poly(dGdC)–silver conjugate using a seed-growing method. A 700 bp poly(dGdC)–Ag+conjugate was reduced with NaBH4 as described in Section 2. The molecules were deposited on mica 10 min after the reduction. NC seeds formed in the DNA template were enlarged by incubating with a mixture of HAuCl4 and ascorbate for 30 s as described in Section 2.6. The inset at the top shows a 1.7 × 1.2 µM selected area. The inset at the bottom provides a statistical analysis of the diameter (corresponding to the measured height) of approximately 300 DNA-associated nanoparticles, showing a relatively narrow distribution with an average height of 2.7 ± 0.6 nm.

To enable the visualization of the extremely small DNA-associated clusters using AFM and TEM, we developed a procedure for their enlargement in the presence of HAuCl_4_ using the weak reductant, ascorbate, following the method described in Section 2. Incubation of the reduced conjugate with a mixture of gold ions and ascorbate resulted in the formation of nanoparticle chains associated with DNA, clearly visible in the AFM images (Figure 8). Incubation of either bare poly(dGdC) or poly(dGdC) conjugated with silver ions did not lead to the formation of DNA-associated particles. The ability to create chains of gold nanoparticles on a DNA template using this approach could be valuable for nanoelectronics applications.

**Figure 9:**
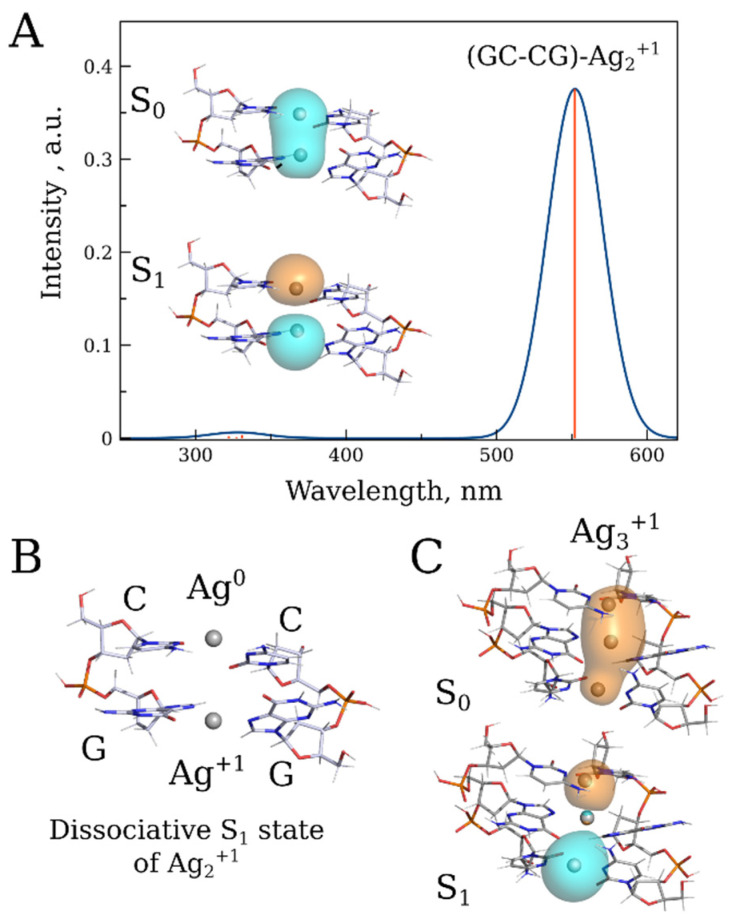
(**A**) Absorption spectrum of the Ag_2_^+1^ associated with two GC base pairs along with a schematic drawing of corresponding S_0_-S_1_ transition NTOs at the PBE0/XTB2 level. (**B**) Geometry of the dissociative S_1_ state of Ag_2_^+1^. (**C**) Illustration of the S_0_-S_1_ transition NTOs of the hypothetical Ag_3_^+1^ excimer emitting at 650 nm.

As shown in Figure S11, the fluorescence emission maximum depends on the length of the conjugate. A noticeable difference in the emission maxima was observed between the 20 bp and 1000 bp conjugates (Figure S12). The maximum in the emission spectrum of the longer conjugate is red-shifted by more than 50 nm compared to that in the spectrum of the 20 bp one. This is likely due to multiple cluster interactions through energy transfer within the long DNA polymer. We utilized DFT/XTB2 calculations to validate the hypothesis regarding cluster interactions derived from experimental results and to explore the potential impact of these interactions on the absorption and fluorescence properties of the conjugate. These calculations provided preliminary insights into the origin of the conjugate’s luminescence. Since the calculated redox potential of the silver atom surrounded by two cytosine bases (E = 2.36 V) is higher than that of the surrounded by two guanine bases (E = 1.46 V), we hypothesized that the preferential oxidation of Ag^o^ by cytosine would lead to an unequal distribution of oxidized and reduced silver within the nucleic acid. Specifically, Ag^o^ would predominantly be surrounded by cytosines, while Ag^+^ would be located near guanines. The absorption spectrum of the Ag^o^–Ag^+^ pair (Ag_2_^+^) associated with two GC base pairs, GC–CG, exhibits an absorption peak with a maximum at 550 nm (Figure 9A). Relaxation of the low-energy excited singlet state of this structure revealed the dissociative nature of the S_1_ state, resulting in a lack of luminescence (Figure 9B) To explain the observed luminescence phenomenon, we propose that upon photodissociation, the ion and atom in the Ag^o^–Ag^+^ pair diverge along the DNA axis in the conjugate, subsequently associating with adjacent non-excited pairs. As a result of this association, two distinct excimer complexes, each composed of three silver atoms/ions, are formed: Ag_3_^+1^ and Ag_3_^+2^. The Ag_3_^+1^ complex can emit photons and is characterized by a band 650 nm in the emission spectrum (Figure 9C), while the Ag_3_^+2^ possesses only one 5s1 electron and does not exhibit distinct bands in the visible range of the spectrum.

## 4. Conclusions

Based on the experimental results and theoretical calculations, we hypothesize that the poly(dGdC)–Ag conjugate functions as a DNA fiber in which silver ions and atoms reversibly interact with each other and with the nucleic bases to form transient silver nanoclusters. Some of these clusters exhibit fluorescent properties, and their interactions through energy transfer result in fluorescence emission of the conjugate at relatively long wavelengths (extending up to 750 nm). The origin and composition of these interacting clusters, as well as the mechanism of their interaction within the DNA, are subjects of ongoing investigation.

## Data Availability

Data are contained within the article and Supplementary Materials.

## Supplementary Materials

The following supporting information can be downloaded at: https://www.mdpi.com/article/10.3390/nano15050397/s1. Figure S1. (a) pdb structure: guanines and cytosines are depicted in green and red, respectively. (b) Optimized structure of the selected representative fragment of the pdb (model 1): the QM region is depicted with tubes (bases) and balls (Ag^+^), while the MM region is represented with lines. Figure S2. (a) 5xjz pdb structure: guanines and cytosines are depicted in green and red, respectively. (b) Optimized structures of the selected representative fragment-1 (model 2) and fragments-2 (model 3) extracted from of the 5xjz pdb. The QM region is depicted with tubes (bases) and balls (Ag^+^), while the MM region is represented with lines. Figure S3. Optimized ground state minima (computed at the QM/MM level) for different models: the QM region is represented with thick sticks for nucleic bases and grey balls for Ag^+^, the backbone is depicted with thin lines. Figure S4. Unshifted CD spectra computed at the QM/MM level of theory for various models are shown: model 1 (green curve), model 2 (red curve), and model 3 (blue curve). For comparison, a dashed black curve represents a GC hexamer without Ag^+^. Figure S5. Structural model 1, optimized at the PCM/M052X level. Figure S6. Computed ECD spectrum for model 1a. PCM/TD-M052X calculations of the spectrum. The spectrum was obtained by broadening each stick contribution using a Gaussian with HWHM = 0.3 eV, and by applying a uniform red-shift of −0.6 eV. Figure S7. (A) Absorption spectra of a 1kbp poly(dGdC) conjugated with Ag^+^ at various DNA (in bp) to Ag+ ratios. A 60 µM (in bp) DNA solution (red curve) was incubated for 10 min at 25 °C with: 20 (green curve), 40 (blue curve), 60 (black curve), and 80 µM (brown curve) AgNO_3_. (B) Differential absorption spectra. The absorption values of the DNA (red curve in A) were subtracted from the corresponding values of the DNA conjugates with 20 (green curve), 40 (blue curve), 60 (black curve) and 80 µM (brown curve) Ag^+^. (C) Dependence of the differential absorption values at 287 nm (panel B) on Ag+ concentration. Figure S8. Statistical height analysis of 1500 bp poly(dGdC) (A) and the DNA conjugate with Ag^+^ (B). The analysis shows that the mean values for both the bare DNA and the conjugate are similar, with average values of 0.9 ± 0.1 nm (A) and 1.01 ± 0.1 nm (B). The analysis was conducted on more than 200 individual molecules. The red curves represent Gaussian fits of the distribution. Figure S9. CD spectra (A) and absorbance spectra (B) of: poly(dGdC) (blue curves) conjugated with Ag^+^ (red) subsequently reduced with NaBH_4_ and measured 5 min after the reduction (green curves). Figure S10. Fluorescence emission spectra (excitation at 620 nm) of poly(dGdC) Ag conjugates 1 day of storage at ambient conditions (red curve) and after 3 months storage at 4 °C (blue curve). The 10% intensity drop is negligible. Figure S11. AFM images and contour length statistics of 700 bp poly(dGdC) molecules conjugated with silver ions and subsequent reduced by BH_4_. The DNA and the reduced conjugate were prepared, deposed on the surface, and scanned by AFM as described in Section 2. The inset shows the statistical contour length analysis of the reduced conjugate. Over 100 single well-separated single molecules were analyzed, yielding an average length of 173 ± 30 nm. Figure S12. Dependence of the fluorescence emission maximum on the DNA length. The conjugates were prepared as described in Section 2.4. Ref. [54] is cited in the Supplementary Materials.

## References

[1] De Heer W.A. (1993). The Physics of Simple Metal Clusters: Experimental Aspects and Simple Models. Rev. Mod. Phys..

[2] Copp S.M., Schultz D., Swasey S., Pavlovich J., Debord M., Chiu A., Olsson K., Gwinn E. (2014). Magic Numbers in DNA-Stabilized Fluorescent Silver Clusters Lead to Magic Colors. J. Phys. Chem. Lett..

[3] Ramazanov R.R., Kononov A.I. (2013). Excitation Spectra Argue for Threadlike Shape of DNA-Stabilized Silver Fluorescent Clusters. J. Phys. Chem. C.

[4] Copp S.M., Schultz D., Swasey S.M., Faris A., Gwinn E.G. (2016). Cluster Plasmonics: Dielectric and Shape Effects on DNA-Stabilized Silver Clusters. Nano Lett..

[5] Petty J.T., Ganguly M., Yunus A.I., He C., Goodwin P.M., Lu Y.-H., Dickson R.M. (2018). A DNA-Encapsulated Silver Cluster and the Roles of Its Nucleobase Ligands. J. Phys. Chem. C.

[6] Wang C., Wang C., Xu L., Cheng H., Lin Q., Zhang C. (2014). Protein-Directed Synthesis of pH-Responsive Red Fluorescent Copper Nanoclusters and Their Applications in Cellular Imaging and Catalysis. Nanoscale.

[7] Xu Y., Sherwood J., Qin Y., Crowley D., Bonizzoni M., Bao Y. (2014). The Role of Protein Characteristics in the Formation and Fluorescence of Au Nanoclusters. Nanoscale.

[8] Sych T.S., Buglak A.A., Reveguk Z.V., Pomogaev V.A., Ramazanov R.R., Kononov A.I. (2018). Which Amino Acids Are Capable of Nucleating Fluorescent Silver Clusters in Proteins?. J. Phys. Chem. C.

[9] Gwinn E., Schultz D., Copp S., Swasey S. (2015). DNA-Protected Silver Clusters for Nanophotonics. Nanomaterials.

[10] Cerretani C., Kanazawa H., Vosch T., Kondo J. (2019). Crystal Structure of a NIR-Emitting DNA-Stabilized Ag _16_ Nanocluster. Angew. Chem. Int. Ed..

[11] Neacşu V.A., Cerretani C., Liisberg M.B., Swasey S.M., Gwinn E.G., Copp S.M., Vosch T. (2020). Unusually Large Fluorescence Quantum Yield for a Near-Infrared Emitting DNA-Stabilized Silver Nanocluster. Chem. Commun..

[12] Kondo J., Tada Y., Dairaku T., Hattori Y., Saneyoshi H., Ono A., Tanaka Y. (2017). A Metallo-DNA Nanowire with Uninterrupted One-Dimensional Silver Array. Nat. Chem..

[13] Müller J. (2019). Nucleic Acid Duplexes with Metal-Mediated Base Pairs and Their Structures. Coord. Chem. Rev..

[14] Scharf P., Müller J. (2013). Nucleic Acids With Metal-Mediated Base Pairs and Their Applications. ChemPlusChem.

[15] Shukla S., Sastry M. (2009). Probing Differential Ag+–Nucleobase Interactions with Isothermal Titration Calorimetry (ITC): Towards Patterned DNA Metallization. Nanoscale.

[16] DiRico D.E., Keller P.B., Hartman K.A. (1985). The Infrared Spectrum and Structure of the Type I Complex of Silver and DNA. Nucleic Acids Res..

[17] Torigoe H., Okamoto I., Dairaku T., Tanaka Y., Ono A., Kozasa T. (2012). Thermodynamic and Structural Properties of the Specific Binding between Ag+ Ion and C:C Mismatched Base Pair in Duplex DNA to Form C–Ag–C Metal-Mediated Base Pair. Biochimie.

[18] Schulze W., Rabin I., Ertl G. (2004). Formation of Light-Emitting Ag _2_ and Ag _3_ Species in the Course of Condensation of Ag Atoms with Ar. ChemPhysChem.

[19] Richards C.I., Choi S., Hsiang J.-C., Antoku Y., Vosch T., Bongiorno A., Tzeng Y.-L., Dickson R.M. (2008). Oligonucleotide-Stabilized Ag Nanocluster Fluorophores. J. Am. Chem. Soc..

[20] Petty J.T., Sergev O.O., Ganguly M., Rankine I.J., Chevrier D.M., Zhang P. (2016). A Segregated, Partially Oxidized, and Compact Ag _10_ Cluster within an Encapsulating DNA Host. J. Am. Chem. Soc..

[21] Obliosca J.M., Babin M.C., Liu C., Liu Y.-L., Chen Y.-A., Batson R.A., Ganguly M., Petty J.T., Yeh H.-C. (2014). A Complementary Palette of NanoCluster Beacons. ACS Nano.

[22] New S.Y., Lee S.T., Su X.D. (2016). DNA-Templated Silver Nanoclusters: Structural Correlation and Fluorescence Modulation. Nanoscale.

[23] Yang M., Zhu L., Yang W., Xu W. (2023). Nucleic Acid-Templated Silver Nanoclusters: A Review of Structures, Properties, and Biosensing Applications. Coord. Chem. Rev..

[24] Gonzàlez-Rosell A., Cerretani C., Mastracco P., Vosch T., Copp S.M. (2021). Structure and Luminescence of DNA-Templated Silver Clusters. Nanoscale Adv..

[25] Yang M., Chen X., Su Y., Liu H., Zhang H., Li X., Xu W. (2020). The Fluorescent Palette of DNA-Templated Silver Nanoclusters for Biological Applications. Front. Chem..

[26] Petty J.T., Story S.P., Juarez S., Votto S.S., Herbst A.G., Degtyareva N.N., Sengupta B. (2012). Optical Sensing by Transforming Chromophoric Silver Clusters in DNA Nanoreactors. Anal. Chem..

[27] Schultz D., Copp S.M., Markešević N., Gardner K., Oemrawsingh S.S.R., Bouwmeester D., Gwinn E. (2013). Dual-Color Nanoscale Assemblies of Structurally Stable, Few-Atom Silver Clusters, As Reported by Fluorescence Resonance Energy Transfer. ACS Nano.

[28] Swasey S.M., Karimova N., Aikens C.M., Schultz D.E., Simon A.J., Gwinn E.G. (2014). Chiral Electronic Transitions in Fluorescent Silver Clusters Stabilized by DNA. ACS Nano.

[29] Karimova N.V., Aikens C.M. (2015). Time-Dependent Density Functional Theory Investigation of the Electronic Structure and Chiroptical Properties of Curved and Helical Silver Nanowires. J. Phys. Chem. A.

[30] Huard D.J.E., Demissie A., Kim D., Lewis D., Dickson R.M., Petty J.T., Lieberman R.L. (2019). Atomic Structure of a Fluorescent Ag _8_ Cluster Templated by a Multistranded DNA Scaffold. J. Am. Chem. Soc..

[31] Leon C., Gonzalez-Abradelo D., Strassert C., Müller J. (2018). Fluorescent DNA-Templated Silver Nanoclusters from Silver(I) Mediated Base Pairs. Chem. Eur.J.

[32] Huang Z., Pu F., Hu D., Chunyan W., Ren J., Xiaogang Q. (2011). Site-Specific DNA-Programmed Growth of Fluorescent and Functional Silver Nanoclusters. Chem. Eur. J.

[33] Kotlyar A.B. (2005). In Vitro Synthesis of Uniform Poly(dG)-Poly(dC) by Klenow Exo- Fragment of Polymerase I. Nucleic Acids Res..

[34] Liu H., Shen F., Haruehanroengra P., Yao Q., Cheng Y., Chen Y., Yang C., Zhang J., Wu B., Luo Q. (2017). A DNA Structure Containing Ag^I^ -Mediated G:G and C:C Base Pairs. Angew. Chem. Int. Ed..

[35] Atsugi T., Ono A., Tasaka M., Eguchi N., Fujiwara S., Kondo J. (2022). A Novel Ag^I^ -DNA Rod Comprising a One-Dimensional Array of 11 Silver Ions within a Double Helical Structure. Angew. Chem. Int. Ed..

[36] Zhao Y., Schultz N.E., Truhlar D.G. (2006). Design of Density Functionals by Combining the Method of Constraint Satisfaction with Parametrization for Thermochemistry, Thermochemical Kinetics, and Noncovalent Interactions. J. Chem. Theory Comput..

[37] Zhao Y., Truhlar D.G. (2008). Density Functionals with Broad Applicability in Chemistry. Acc. Chem. Res..

[38] Andrae D., Hubermann U., Dolg M., Stoll H., Preu H. (1990). Energy-Adjusted Ab Initio Pseudopotentials for the Second and Third Row Transition Elements. Theor. Chim. Acta.

[39] Tomasi J., Mennucci B., Cammi R. (2005). Quantum Mechanical Continuum Solvation Models. Chem. Rev..

[40] Martínez Fernández L., Santoro F., Improta R. (2022). Nucleic Acids as a Playground for the Computational Study of the Photophysics and Photochemistry of Multichromophore Assemblies. Acc. Chem. Res..

[41] Improta R., Santoro F., Blancafort L. (2016). Quantum Mechanical Studies on the Photophysics and the Photochemistry of Nucleic Acids and Nucleobases. Chem. Rev..

[42] Katrivas L., Makarovsky A., Kempinski B., Randazzo A., Improta R., Rotem D., Porath D., Kotlyar A.B. (2024). Ag+-Mediated Folding of Long Polyguanine Strands to Double and Quadruple Helixes. Nanomaterials.

[43] Martínez-Fernández L., Kohl F.R., Zhang Y., Ghosh S., Saks A.J., Kohler B. (2024). Triplet Excimer Formation in a DNA Duplex with Silver Ion-Mediated Base Pairs. J. Am. Chem. Soc..

[44] Frisch M.J., Trucks G.W., Schlegel H.B., Scuseria G.E., Robb M.A., Cheeseman J.R., Scalmani G., Barone V., Petersson G.A., Nakatsuji H. (2016). Gaussian 16.

[45] Gell L., Kulesza A., Petersen J., Röhr M.I.S., Mitrić R., Bonačić-Koutecký V. (2013). Tuning Structural and Optical Properties of Thiolate-Protected Silver Clusters by Formation of a Silver Core with Confined Electrons. J. Phys. Chem. C.

[46] Liasi Z., Jensen L., Mikkelsen K.V. (2024). A Combined Quantum Mechanics and Molecular Mechanics Approach for Simulating the Optical Properties of DNA-Stabilized Silver Nanoclusters. J. Chem. Theory Comput..

[47] Adamo C., Barone V. (1999). Toward Reliable Density Functional Methods without Adjustable Parameters: The PBE0 Model. J. Chem. Phys..

[48] Hellweg A., Hättig C., Höfener S., Klopper W. (2007). Optimized Accurate Auxiliary Basis Sets for RI-MP2 and RI-CC2 Calculations for the Atoms Rb to Rn. Theor. Chem. Acc..

[49] Bannwarth C., Ehlert S., Grimme S. (2019). GFN2-xTB—An Accurate and Broadly Parametrized Self-Consistent Tight-Binding Quantum Chemical Method with Multipole Electrostatics and Density-Dependent Dispersion Contributions. J. Chem. Theory Comput..

[50] Ehlert S., Stahn M., Spicher S., Grimme S. (2021). Robust and Efficient Implicit Solvation Model for Fast Semiempirical Methods. J. Chem. Theory Comput..

[51] Neese F., Wennmohs F., Becker U., Riplinger C. (2020). The ORCA Quantum Chemistry Program Package. J. Chem. Phys..

[52] Shimodaira Y., Miura T., Kudo A., Kobayashi H. (2007). DFT Method Estimation of Standard Redox Potential of Metal Ions and Metal Complexes. J. Chem. Theory Comput..

[53] Zikich D., Lubitz I., Alexander K. (2010). Ag+ Induced Arrangement of Poly(dC) into Compact Ring-Shaped Structures. Int. Rev. Biophys. Chem..

[54] Avila Ferrer F.J., Cerezo J., Stendardo E., Improta R., Santoro F. (2013). Insights for an Accurate Comparison of Computational Data to Experimental Absorption and Emission Spectra: Beyond the Vertical Transition Approximation. J. Chem. Theory Comput..

