# Supplementary material for: Fluorescent Silver Nanoclusters Associated with Double-Stranded Poly(dGdC) DNA
Source: Nanomaterials (Basel). 2025 Mar 5;15(5):397. doi: 10.3390/nano15050397 (PMC11902164; doi:10.3390/nano15050397)
Supplement: Supplementary file 1 [file nanomaterials-15-00397-s001.zip › nanomaterials-3507470-supplementary.pdf]

Fluorescent Silver Nanoclusters Associated with Double-Stranded Poly(dGdC) DNA

Zakhar Reveguk, Roberto Improta, Lara Martínez-Fernández, Ruslan Ramazanov, Shachar Richter and Alexander Kotlyar

SUPPORTING INFORMATION

Quantum mechanical calculations of CD spectra

In addition to the QM/MM approach described in the main text, we verified our conclusion by performing full QM calculations on one of the structural models described below (model 1a). These calculations utilized the M052X functional, with the LANL2DZ pseudopotential for Ag^{+} ions and the 6-31+G(d,p) basis set for the remaining atoms. Solvent effects are included with the PCM model.

Simulation of CD spectra.
To simulate the ECD spectra, we computed the excitation energies for the 30-40 lowest energy excited states (depending on the model size) Each transition was then broadened with a Gaussian with half width at half maximum (HWHM) of 0.3 eV. The lack of vibrational and thermal effects in our calculations, resulted in a systematic blue-shift in the computed vertical transition energies compared to the maximum of the absorption band[1]. Consequently, the computed spectra are expected to be blue-shifted with respect to the experimental ones. To facilitate comparison with experiments results, we have red-shifted the computed spectra by 0.6 eV, a value consistent with the difference observed when comparing the experimental absorption spectra of dG and dC with those computed using the same approach. The un-shifted spectra are shown in Fig.S4.

1. Computational models

For geometry optimizations, we have considered two different initial structures[1], using pdb files 7xkm and 5xjz. From 7xkm pdb (Fig.S1a) we have extracted a representative fragment (Fig. S1b) containing 2 C-Ag-C steps, one G-Ag-C step and a terminal G, referred to as $(CGC)_2G$-3Ag). We label this structure as model 1. The 5xjz pdb is quite unique; it comprises two different fragments. One fragment is formed by C-Ag-C and G-Ag-G pairs (framed in black in Fig. S2a, fragment-1) and the other contains two types of "tetrads" formed by G and C (framed in yellow in Fig. S2a, fragment-2). We label these structures as model 2 and model 3, respectively. All these models are illustrated in Fig.S3.

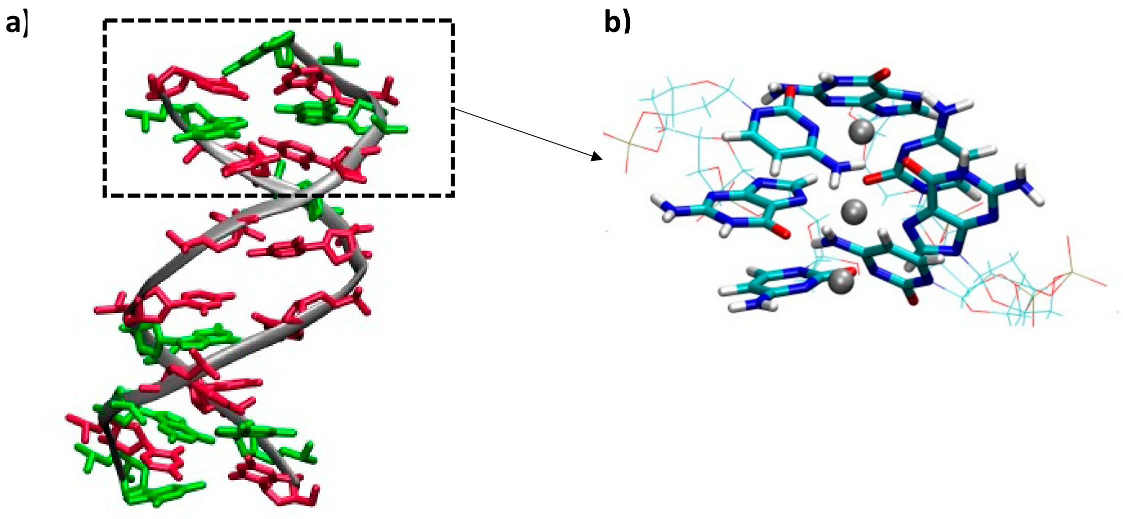

Figure S1. a) pdb structure: guanines and cytosines are depicted in green and red, respectively. b) Optimized structure of the selected representative fragment of the pdb (model 1): the QM region is depicted with tubes (bases) and balls (Ag^{+}), while the MM region is represented with lines.

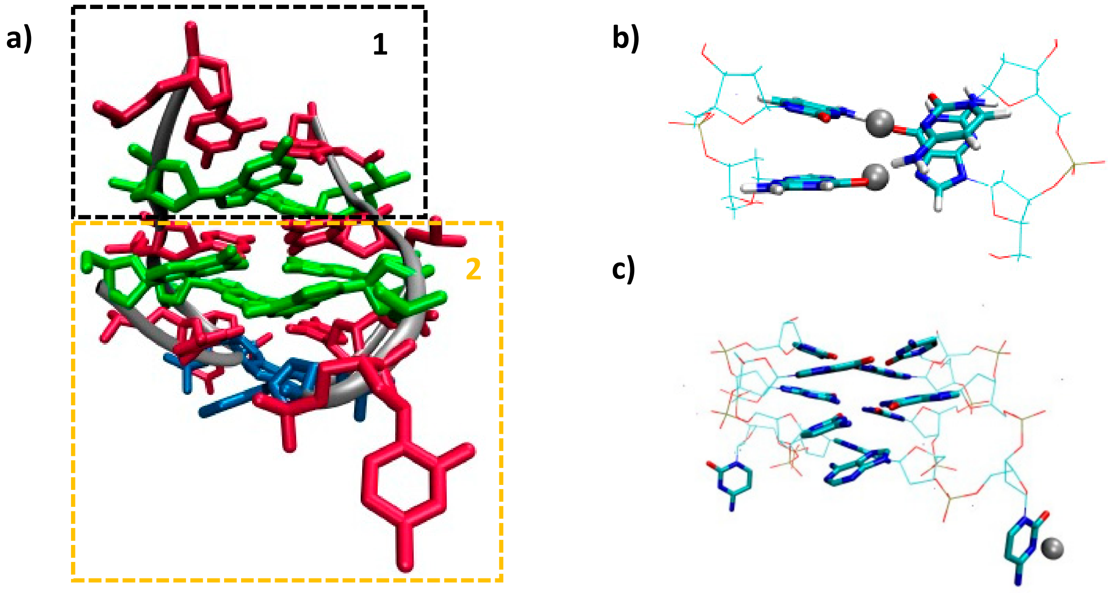

Figure S2. a) 5xjz pdb structure: guanines and cytosines are depicted in green and red, respectively. b) Optimized structures of the selected representative fragment-1 (model 2) and fragments-2 (model 3) extracted from of the 5xjz pdb. The QM region is depicted with tubes (bases) and balls (Ag^{+}), while the MM region is represented with lines.

2. Calculation of ECD spectra.

We computed the ECD spectra for the three structural models using QM/MM calculations.

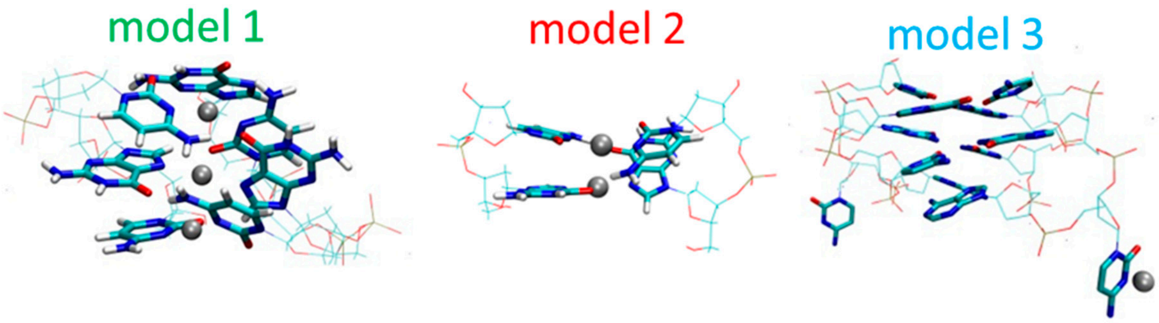

Figure S3. Optimized ground state minima (computed at the QM/MM level) for different models: the QM region is represented with thick sticks for nucleic bases and grey balls for Ag^+, the backbone is depicted with thin lines.

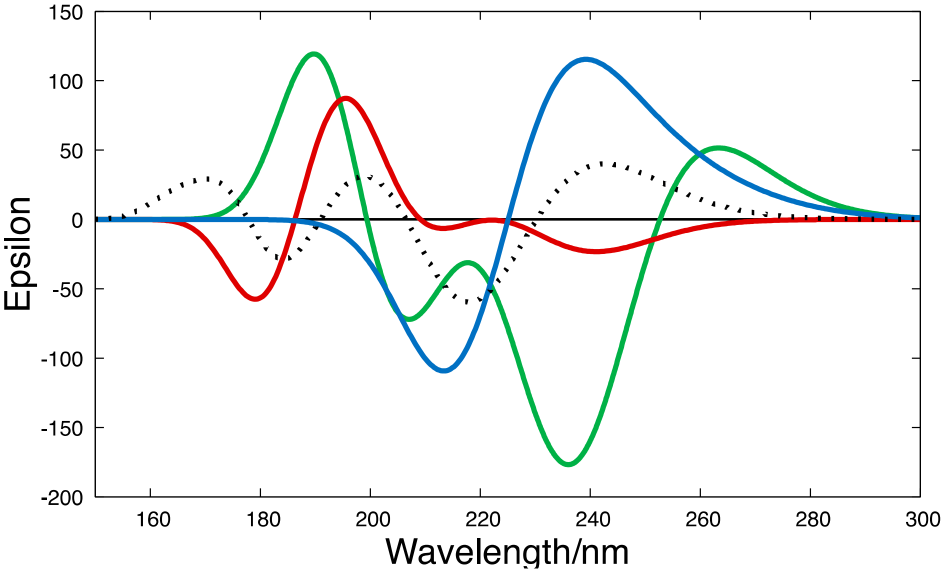

Figure S4. Unshifted CD spectra computed at the QM/MM level of theory for various models are shown: model 1 (green curve), model 2 (red curve), and model 3 (blue curve). For comparison, a dashed black curve represents a GC hexamer without Ag^+

The experimental CD spectrum exhibits a positive band around 300 nm followed by a strong negative band below 290 nm, featuring two distinct minima. The predicted spectral lineshape for model 1 closely matches the experimental data. On the other hand, the spectra computed for models 2 and 3 (extracted from 5xjz.pdb) differ significantly from the experimental one. Our calculations thus suggest that the structural arrangement of GC-composed nucleic acid molecules in the presence of Ag^+ resembles that found in 7xkm.pdb, characterized by C-Ag-C and G-Ag-G pairs. To further validate this conclusion and determine if the addition 'dangling' G residue present in model 1 is necessary to produce the experimental ECD spectrum, we investigated the structure depicted in Fig. S5 (model 1a). This structure includes only six bases (2 CC and 1 GG pair) each coordinating with Ag^+.

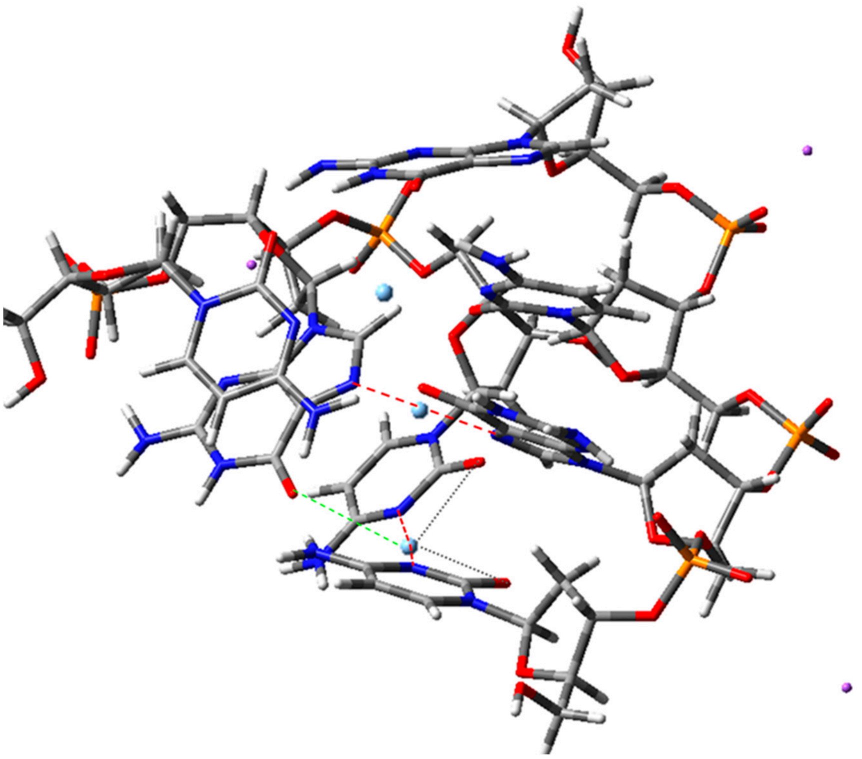

Figure S5. Structural model 1, optimized at the PCM/M052X level.

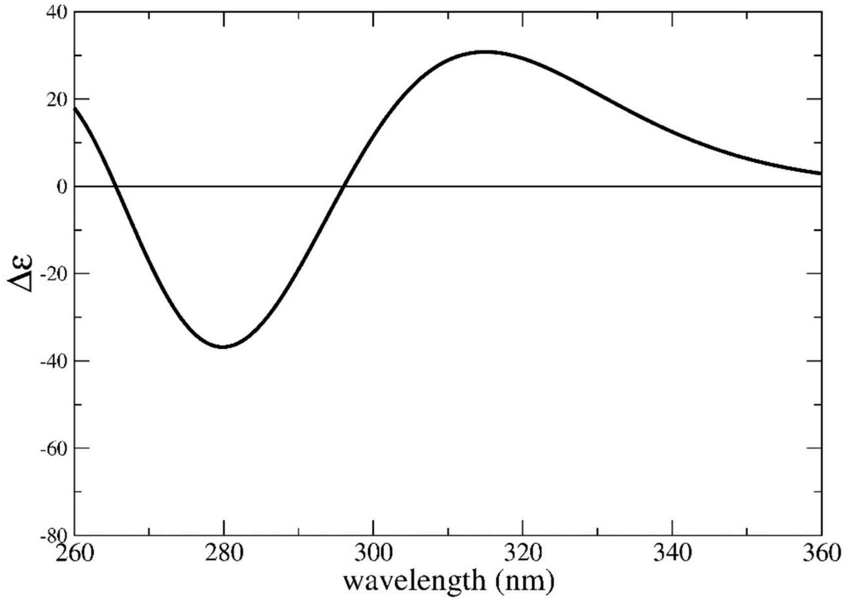

Figure S6. Computed ECD spectrum for model 1a. PCM/TD-M052X calculations of the spectrum. The spectrum was obtained by broadening each stick contribution using a Gaussian with HWHM=0.3 eV, and by applying a uniform red-shift of -0.6 eV.

The computed ECD spectrum (Fig. S6) successfully reproduces the main features of the experimental spectrum, of the DNA- Ag^{+} conjugate, providing further evidence for the validity of structural models 1 and 1a.

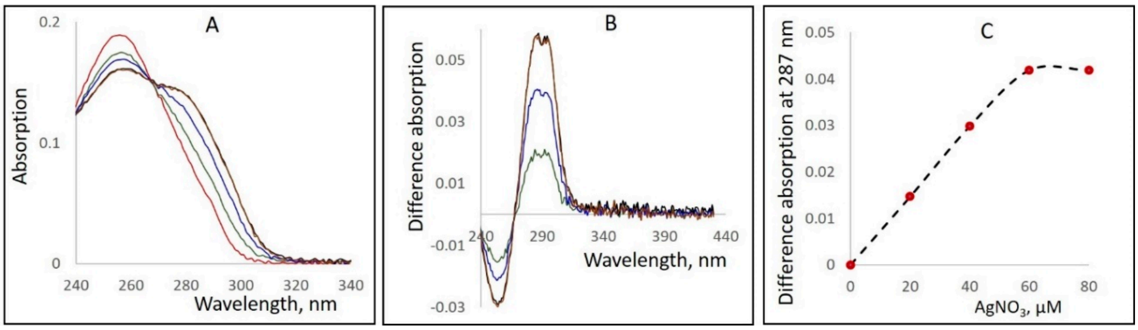

Figure S7. (A) Absorption spectra of a 1kbp poly(dGdC) conjugated with Ag^+ at various DNA (in bp) to Ag+ ratios. A 60 µM (in bp) DNA solution (red curve) was incubated for 10 min at 25 °C with: 20 (green curve), 40 (blue curve), 60 (black curve), and 80 µM (brown curve) $AgNO_3$. (B) Differential absorption spectra. The absorption values of the DNA (red curve in A) were subtracted from the corresponding values of the DNA conjugates with 20 (green curve), 40 (blue curve), 60 (black curve) and 80 µM (brown curve) Ag^+. (C) Dependence of the differential absorption values at 287 nm (panel B) on Ag+ concentration.

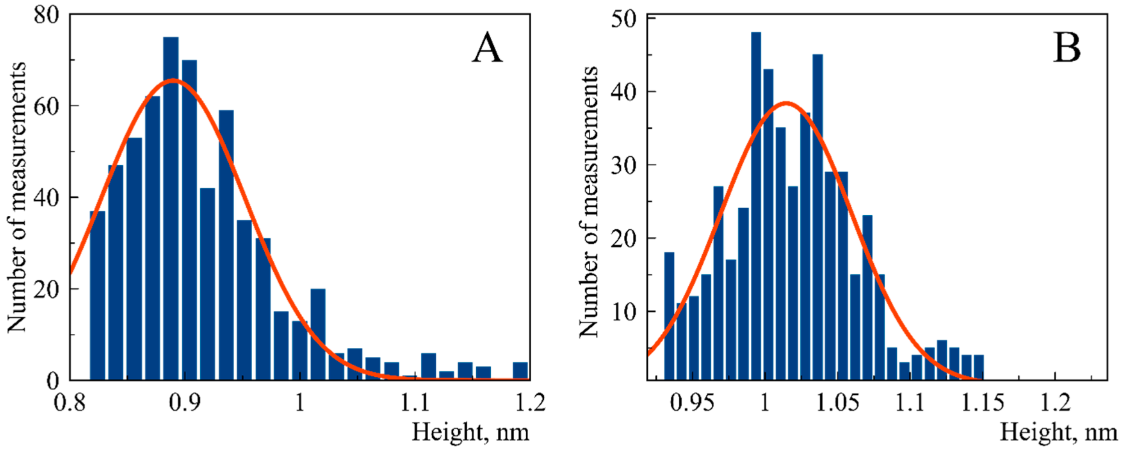

Figure S8. Statistical height analysis of 1500 bp poly(dGdC) (A) and the DNA conjugate with Ag^+ (B). The analysis shows that the mean values for both the bare DNA and the conjugate are similar, with average values of 0.9 ± 0.1 nm (A) and 1.01± 0.1 nm (B). The analysis was conducted on more than 200 individual molecules. The red curves represent Gaussian fits of the distribution.

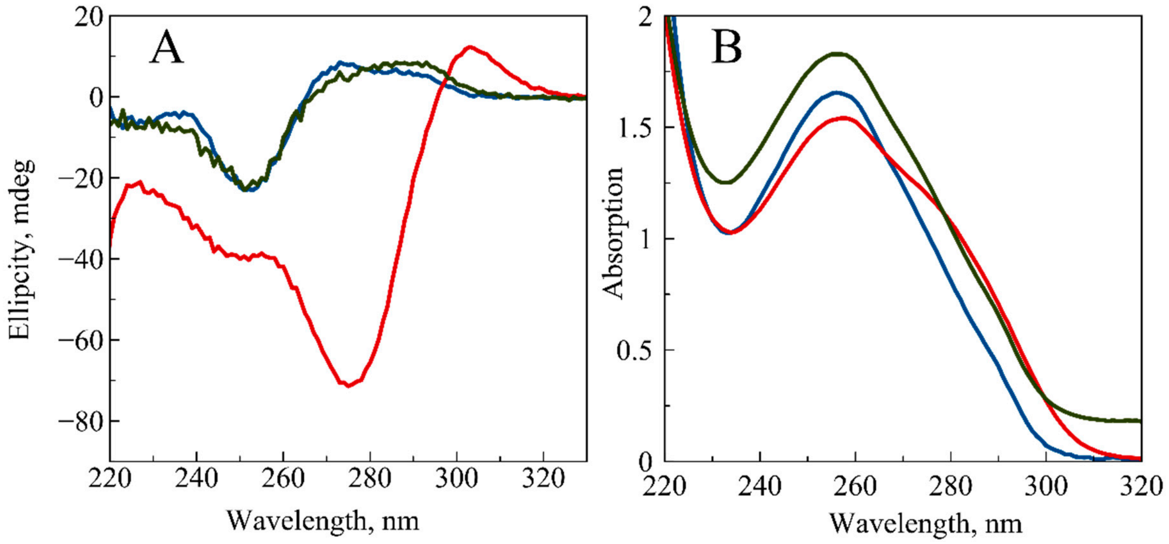

Figure S9. CD spectra (A) and absorbance spectra (B) of: poly(dGdC) (blue curves) conjugated with Ag^+ (red) subsequently reduced with $NaBH_4$ and measured 5 min after the reduction (green curves).

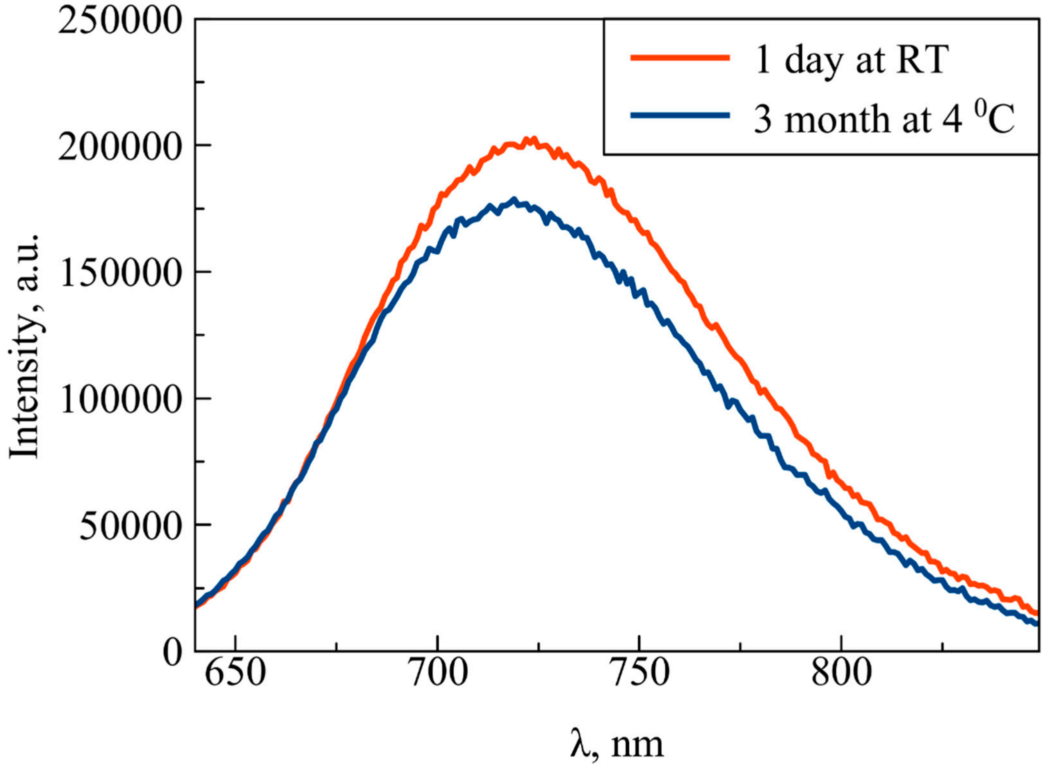

Figure S10. Fluorescence emission spectra (excitation at 620 nm) of poly(dGdC) Ag conjugates 1 day of storage at ambient conditions (red curve) and after 3 months storage at 4 ^{0}C (blue curve). The 10% intensity drop is negligible.

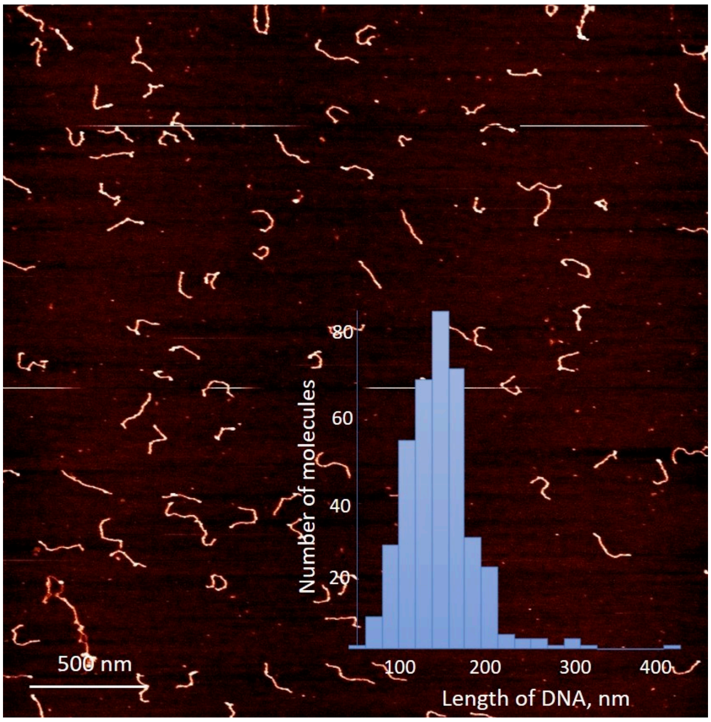

Figure S11. AFM images and contour length statistics of 700 bp poly(dGdC) molecules conjugated with silver ions and subsequent reduced by BH_4. The DNA and the reduced conjugate were prepared, deposed on the surface, and scanned by AFM as described in Materials and Methods. The inset shows the statistical contour length analysis of the reduced conjugate. Over 100 single well-separated single molecules were analyzed, yielding an average length of 173 ± 30 nm.

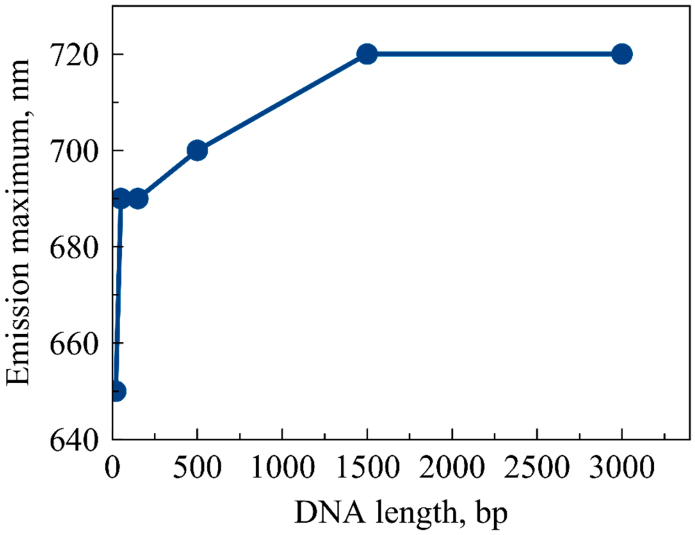

Figure S12. Dependence of the fluorescence emission maximum on the DNA length. The conjugates were prepared as described in Materials and Methods (section 2.4).

References:

(1) Avila Ferrer, F. J.; Cerezo, J.; Stendardo, E.; Improta, R.; Santoro, F. Insights for an Accurate Comparison of Computational Data to Experimental Absorption and Emission Spectra: Beyond the Vertical Transition Approximation. *J. Chem. Theory Comput.* **2013**, *9* (4), 2072–2082. https://doi.org/10.1021/ct301107m.
